# Primary care clinic visits in formerly evacuated areas due to radiation disaster following the Great East Japan Earthquake: A retrospective descriptive study

**DOI:** 10.1097/MD.0000000000037942

**Published:** 2024-05-03

**Authors:** Saori Nonaka, Masaaki Odaka, Akemi Takada, Yuki Senoo, Toyoaki Sawano, Akihiko Ozaki, Michio Murakami, Makoto Yoshida, Yuna Uchi, Katsuko Onoda, Tomoyoshi Oikawa, Masaharu Tsubokura

**Affiliations:** a Department of Radiation Health Management, Fukushima Medical University School of Medicine, Fukushima, Japan; b Research Center for Community Health, Minamisoma Municipal General Hospital, Fukushima, Japan; c Department of General Medicine, Taito Hospital, Japan Association for Development of Community Medicine, Tokyo, Japan; d Clinic Director, Odaka Clinic Affiliated with Minamisoma Municipal General Hospital, Fukushima, Japan; e Department of Nursing, Odaka Clinic Affiliated with Minamisoma Municipal General Hospital, Fukushima, Japan; f Department of Internal Medicine, Higashi-Totsuka Memorial Hospital, Kanagawa, Japan; g Department of Surgery, Jyoban Hospital of Tokiwa Foundation, Fukushima, Japan; h Department of Breast and Thyroid Surgery, Jyoban Hospital of Tokiwa Foundation, Fukushima, Japan; i Department of Health Risk Communication, Fukushima Medical University School of Medicine, Fukushima, Japan; j Center for Infectious Disease Education and Research (CiDER), Osaka University, Osaka, Japan (current address); k Department of Nursing, Minamisoma Municipal General Hospital, Fukushima, Japan; l Department of Neurosurgery, Minamisoma Municipal General Hospital, Fukushima, Japan.

**Keywords:** disaster planning, Fukushima nuclear accident, health services needs and demand, medical records, primary health care

## Abstract

Radiation disasters pose distinctive medical challenges, requiring diverse care approaches. Beyond radiation exposure assessment, addressing health impacts due to lifestyle changes, especially among vulnerable populations, is vital. Evacuation orders issued in radiation-affected areas introduce unique healthcare dynamics, with their duration significantly influencing the recovery process. Understanding evolving patient demographics and medical needs after lifting evacuation orders is crucial for post-disaster care planning. Minamisoma Municipal Odaka Hospital, located 13 to 20 km from Fukushima Daiichi Nuclear power plant in a post-evacuation zone, was greatly affected by the Great East Japan Earthquake and subsequent radiation disaster. Data were retrospectively collected from patient records, including age, gender, visit date, diagnoses, and addresses. Patient records from April 2014 to March 2020 were analyzed, comparing data before and after the July 2016 evacuation order lift. Data was categorized into pre and post-evacuation order lifting periods, using International Classification of Diseases, Tenth Edition codes, to identify the top diseases. Statistical analyses, including χ-square tests, assessed changes in disease distributions. Population data for Odaka Ward and Minamisoma City fluctuated after lifting evacuation orders. As of March 11, 2011, Odaka Ward had 12,842 residents (27.8% aged 65+ years), dropping to 8406 registered residents and 2732 actual residents by April 30, 2018 (49.7%). Minamisoma City also saw declines, with registered residents decreasing from 71,561 (25.9%) to 61,049 (34.1%). The study analyzed 11,100 patients, mostly older patients (75.1%), between 2014 and 2020. Post-lifting, monthly patient numbers surged from an average of 55.2 to 213.5, with female patients increasing from 33.8% to 51.7%. Disease patterns shifted, with musculoskeletal cases declining from 23.8% to 13.0%, psychiatric disorders increasing from 9.3% to 15.4%, and trauma-related cases decreasing from 14.3% to 3.9%. Hypertension (57.1%) and dyslipidemia (29.2%) prevailed post-lifting. Urgent cases decreased from 1.3% to 0.1%. This study emphasizes the importance of primary care in post-evacuation zones, addressing diverse medical needs, including trauma, noncommunicable diseases, and psychiatric disorders. Changing patient demographics require adaptable healthcare strategies and resource allocation to meet growing demands. Establishing a comprehensive health maintenance system tailored to these areas’ unique challenges is crucial for future disaster recovery efforts.

## 1. Introduction

Various types of medical care are required during radiation disaster recovery. In addition to the assessment of internal and external radiation exposure to the population,^[1–4]^ the health effects caused by their life changes (e.g., worsening of chronic and mental illnesses, psychological effects, and problems in care and welfare among the older, disabled, and other vulnerable groups) must be appropriately addressed using a limited medical supply system.^[5–10]^

One of the characteristics of a radiation disaster is that radiation doses create off-limits areas, for which evacuation orders are issued.^[11]^ The radiation dose determines how quickly people can return to their original locations after a radiation disaster. In areas where people return quickly, medical facilities can be easily restored; however, in areas where evacuation is prolonged, it is more difficult because most of their functions are lost due to the evacuation.

In areas where evacuation orders are issued due to radiation disasters, the types of people who stay in the area will change. Once an evacuation order is issued, all people, including medical facilities and public institutions, cease to travel to the area for a significant period of time. Subsequently, to reconstruct the affected areas, the number of residents and workers involved in nuclear accident clean-up gradually increase in number. Eventually, residents begin to return home, and the patient population and demand for medical care may differ from that observed during the recovery period of other disasters.

Although several radiation disasters have occurred in the past, there have been only a limited number of cases where evacuation orders were subsequently lifted and residents returned to their homes. Notably, after the Chornobyl accident in 1986, evacuation orders were not lifted; thus, a small number of residents who could not adapt to their new location and were attached to their familiar surroundings returned home, who called “self-settlers.”^[12,13]^ Unlike the Chernobyl accident, in the Great East Japan Earthquake (GEJE) and subsequent radiation disaster in 2011, air doses in Minamisoma were low and residents were able to return early.^[14–17]^ Although there are some reports of emergency medical systems in evacuation areas after the disaster,^[18–20]^ there are no reports of usual medical visits before and after the lifting of the evacuation order. The reopening of medical facilities is a top priority for residents when making decisions about returning to their homes.^[21,22]^ More multifaceted information on the demand for medical care in areas where evacuation orders have been lifted due to radiation disaster, will also contribute to planning what medical care should be provided when evacuation orders are lifted.

In this study, we examined the medical records of patients who visited Minamisoma Municipal Odaka Hospital (Odaka Clinic affiliated with Minamisoma Municipal General Hospital in August 2019), which resumed medical services in Odaka Ward, Minamisoma City, Fukushima Prefecture, an evacuation-ordered zone, with the aim to determine the medical demand before and after the evacuation order was lifted. Further, this study aimed to describe the demand for medical care after a radiation disaster and examine key factors for future radiation disaster recovery processes.

## 2. Materials and methods

### 2.1. Backgrounds of Odaka ward and Odaka hospital

**Figure 1.**
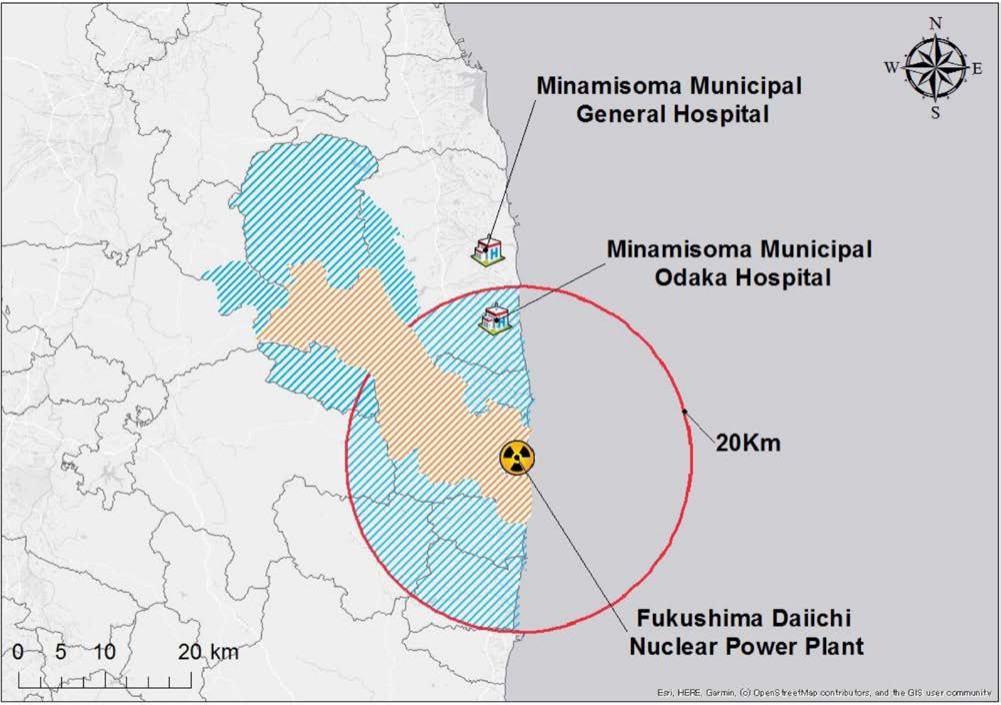
The study-related location information illustrates the difficult-to-return zones, evacuation order lifted zones, FDNPP, and the locations of municipal hospitals.

Odaka Ward, Minamisoma City, is located approximately 13 to 20 km from the Fukushima Daiichi Nuclear power plant (Fig. 1). Preceding the occurrence of the GEJE, the region accommodated approximately 13,000 residents and was endowed with a total of 14 medical establishments, which encompassed 7 clinics, 5 dental clinics, and 2 hospitals, one of which was the Minamisoma Municipal Odaka Hospital (Odaka Hospital). These clinics offered specialized services in internal medicine, dermatology, orthopedics, and surgical interventions. One of the 2 hospitals operated as a dedicated psychiatric care facility.

Table 1 shows the timeline of events and conditions of the FNDPP and Odaka Hospital following the GEJE. On March 11, 2011, the Tohoku-Pacific Ocean Earthquake struck, and on March 12, 2011, an evacuation order was issued within a 20-km radius of Tokyo Electric Power Co., Inc. Fukushima Daiichi Nuclear power plant. This led to the evacuation of all residents and closure of medical facilities in Odaka Ward, making Odaka Ward inaccessible, with the exception of passing through to the nuclear power plant. On July 12, 2016, the evacuation order was lifted, and residents began to return to their homes. As of March 2020, 3663 residents had returned, and 3 clinics were providing medical services. One is the Odaka hospital and the others resumed in April 2016. After the return, the external radiation exposure of the residents of Odaka Ward was kept low.^[23]^

**Table 1:** Timeline of events and condition of the nuclear power plants and Odaka Hospital following the earthquake.

Date	Time	Happened in FDNPP, administrative instructions/★ In Odaka Hospital
March 11, 2011	14:11	The Great East Japan Earthquake occurred.
	19:03	The Japanese government issued a declaration of nuclear emergency for the FDNPP.
	21:23	Evacuation order was issued for residents within 3 km of the FDNPP.
March 12, 2011	3:51	Evacuation order was extended to the area within a 3–10 km radius.
	7:45	The Japanese government extended the nuclear emergency declaration to the FDNPP
		3109 people were evacuated to public shelters in Odaka Ward.
	15:36	Hydrogen explosion at Unit 1
		★ Transferred inpatients to MMGH and closed since then
	18:25	Evacuation order was extended to the area within 20 km of the FDNPP.
March 13, 2011		Public evacuation centers in Odaka Ward were closed.
March 14, 2011	11:01	NISA called for 600 residents within a 20 km radius of the FDNPP to shelter indoors.
	11:01	Hydrogen explosion at Unit 3
March 15, 2011	6:14	Hydrogen explosion at Unit 4
	11:08	Indoor evacuation order for approximately 140,000 residents and others within 20–30 km of the FDNPP.
March 25, 2011	11:46	The government requested municipalities within a 20–30 km radius of the FDNPP to evacuate residents voluntarily.
April 22, 2011		The evacuation order within 20–30 km of the FDNPP was lifted. The area within a 20 km radius was designated as an “Evacuation order zone” and off-limits. Areas outside the 20 km radius of the FDNPP had a high cumulative amount of radioactive materials and were designated as “Planned Evacuation Zones,”; most of the areas outside the 20–30 km radius that did not fall within the Planned Evacuation Zones were designated as “Emergency Evacuation Preparation Zones.”
May 25, 2011		Furloughs in Odaka Ward began with a permit system.
July, 2011		Minamisoma City started its decontamination work.
September 30, 2011		“Emergency Evacuation Preparation Zones (5 municipalities)” were lifted.
April 16, 2012		The areas within a radius of 20 km were reclassified as the “Evacuation order cancelation preparation zone” (20 mSv/yr or less), the “Restricted residence zone” (more than 20 mSv/yr but less than 50 mSv/yr), and the “Difficult-to-Return zone” (more than 50 mSv/yr). Except for the Difficult-to-Return zones, it was possible in other zones to stay and conduct restoration activities that do not require an overnight stay.
January, 2012		The government began advanced decontamination of particular decontamination areas.
Augst 26, 2013		The government began decontamination in Minamisoma City.
April 23, 2014		★Reopened and opened 3 days a week.
July 1, 2015		★Opened 4 days a week
April 1, 2016		★Open every weekday
July 12, 2016		The Evacuation order cancelation preparation zone and Restricted residence zone were lifted, and the area became habitable.
March 31, 2017		Government decontamination completed.
April 1, 2017		★Home medical care (home-visit medical care and online medical care) began.

FDNPP = Fukushima Daiichi Nuclear Power Plant, MMGH = Minamisoma Municipal General Hospital, NISA = The Nuclear and Industrial Safety Agency.

Odaka Hospital, the setting for this study, was a municipal hospital with 99 inpatient beds and outpatient services before the GEJE. Following the evacuation order issued on March 12, all inpatients and staff were moved to Minamisoma Municipal General Hospital (Haramachi Ward) on March 13. Subsequently, all medical services were suspended; however, in anticipation of resident return, the “Minamisoma City Odaka Ward Regional Medical Reconstruction Plan” was formulated in 2013.^[21]^ The Odaka Hospital was partially restored, and outpatient insurance treatment resumed in April 2014. In August 2019, the hospital was moved from its previous location (3-8 Higashimachi, Odaka-ku, Odaka) to a new location within the Odaka Health Center (84 Odaka Kanayamae, Odaka-ku, Odaka), which was approximately 200 m away from its previous location and provides outpatient care every weekday.

### 2.2. Universal health coverage in Japan

Japan has achieved universal health coverage by creating both employee-based and community-based health social insurance.^[24]^ Most hospitals provide medical care covered by health insurance, including the Odaka Hospital. The Japanese health insurance system covers most medical procedures for treatment purposes. Therefore, aggregating the records of insurance care at the Odaka Hospital reflects the demand for medical care in this area.

### 2.3. Research design

This study was a retrospective descriptive study. In Odaka Hospital, data on medical records was extracted retrospectively, from patients seen between April 2014 (when Odaka Hospital resumed outpatient services) and March 2020, to determine which patients were seen at local medical institutions before and after the evacuation order was lifted.

This study conforms with the Strengthening the Reporting of Observational Studies in Epidemiology guidelines.

### 2.4. Data collection

The residency data for 2011 and 2018–2021 in Odaka Ward and Minamisoma City were provided by the Minamisoma City Hall. This data included the number of registered residents and the number of actual residents (including the number of residents over 65 years old).

The medical records from April 2014 to March 2020 (for insured patients) included age, sex, year and month of visit, disease name, and patient address (as written on the insurance card or as reported at the time of medical treatment for patients.

The residency data for 2011 and 2018–2021 in Odaka Ward and Minamisoma City were provided by the Minamisoma City Hall. This data included the number of registered residents and the number of actual residents (including the number of residents over 65 years old).

The medical records from April 2014 to March 2020 (for insured patients) included age, sex, year and month of visit, disease name, and patient address (as written on the insurance card or as reported at the time of medical treatment for patients outside the Fukushima Prefecture). For patients outside the Fukushima Prefecture, the address was provided to the municipality (for those in Minamisoma City, the address was provided to the ward name), along with information related to whether the patient was transported to the emergency room and whether the patient received a first visit or following treatment. “First visit” means the first consultation in this research period. If they had visited Odaka Hospital before the earthquake, that visit was not taken into consideration. We didn’t have any missing data.

The data were also divided into 2 major categories: before and after the evacuation order was lifted in in July 2016, and the top 20 diseases in each category were tabulated. Disease classification was based on the International Classification of Diseases, Tenth Edition (ICD-10), and ICD-10 codes, disease names, and number of diseases were compiled.

All data were entered into Microsoft Excel for Mac Version 16.75.2 (Microsoft Corporation, Redmond, WA).

The data used in this study are not available to the public because the Odaka Hospital provided for the sole use of this research study due to the inclusion of personal information in the data.

### 2.5. Statistical analysis

Each column represents the proportion of patients with specific diseases out of the total number of patients observed during the total number of patients in the respective period. The percentage of patients diagnosed with musculoskeletal (ICD-10: M code), psychiatric (F code), and Injury, poisoning, and other consequences of external causes (S and T codes) was calculated in relation to the total number of patients, for both the pre- and post-evacuation order lifting periods.

χ-square tests were performed on the percentages of patients with musculoskeletal (ICD-10: M code), psychiatric (F code), and diseases due to trauma or external causes (S and T code) before and after the lifting of the evacuation order, respectively. A *P* value of < 0.05 was considered statistically significant.

### 2.6. Ethical consideration

This study was approved by the Ethics Committees of the Minamisoma Municipal General Hospital (approval number: 3-09) and Fukushima Medical University (approval number: 2020-171). In addition, the study was publicized in writing, and an opt-out procedure was implemented to guarantee participants the opportunity to refuse consent.

## 3. Results

### 3.1. Residents of Odaka Ward and Minamisoma city as a whole

Table 2 lists the number of registered residents and the number of actual residents, the percentage of actual residents to the number of registered residents, the number of persons aged 65 and over, and the aging rate by year for Odaka Ward and the entire Minamisoma City including Odaka Ward, respectively. As of March 11, 2011, 12,842 residents (3575 aged ≥ 65 years, or aging rate 27.8%) were registered in Odaka Ward. By April 30, 2018, the year after the evacuation order was lifted, the number of registered residents had decreased to 8406, and the number of actual residents was 2732 (1359, 49.7%). The reason for the difference between the number of registered residents and the actual number of residents is thought to be that some residents are registered but reside in other areas. Resident registration then continued to decline through 2021, with 6925 residents (2992, 43.2%) on March 31, 2021. Meanwhile, the number of actual residents increased to 3752 (1854, 49.4%).

**Table 2:** People living in Odaka and Minamisoma city.

	2011/3/11	2016/7/31	2017/3/31	2018/3/31	2018/4/30	2019/3/31	2020/3/31	2021/3/31
Odaka Ward								
Number of registered residents	12,842	9778	9079	8412	8406	7785	7290	6925
Number of >64 years old	3575	―	―	―	―	―	3015	2992
Aging rate (%)	27.8	―	―	―	―	―	41.4	43.2
Number of actual residences	―	310	1487	2639	2732	3491	3663	3752
Number of >64 years old	―	183	799	1327	1359	1725	1806	1854
Aging rate (%)	―	59.0	53.7	50.3	49.7	49.4	49.3	49.4
Actual residents to registrations (%)	―	3.2	16.4	31.4	32.5	44.8	50.2	54.2
Minamisoma city including Odaka Ward								
Number of registered residents	71,561	63,355	62,298	61,000	61,049	60,197	59,377	58,574
Number of >64 years old	18,547	―	―	―	20,812	20,968	21,053	21,234
Aging rate (%)	25.9	―	―	―	34.1	34.8	35.5	36.3
Number of actual residents	―	53,420	53,917	54,270	54,487	54,505	54,542	54,394
Number of >64 years old	―	18,070	18,561	19,095	19,134	19,463	19,682	19,965
Aging rate (%)	―	33.8	34.4	35.2	35.1	35.7	36.1	36.7
Actual residents to registrations (%)	―	84.3	86.5	89.0	89.3	90.5	91.9	92.9

In Minamisoma City as a whole, 71,561 residents (18,547 aged 65 years and over; aging rate 25.9%) were registered as of March 11, 2011. By April 30, 2018, the year after the evacuation order for Odaka Ward was lifted, the number of registered residents had decreased to 61,049 (20,812, 34.1%), leaving 54,487 (19,134, 35.1%) actually living in the area. Resident registration then continued to decline through 2021, reaching 58,574 (21,234, 36.3%) on March 31, 2021. Meanwhile, the actual number of residents was 54,394 (19,965, 36.7%).

### 3.2. Patient information

Table 3 summarizes the patients who visited Odaka Hospital from April 2014 to March 2020, before and after the evacuation order was lifted, and by year, patients’ basic information, and Table 4 shows the ranking of the top disease names (ICD-10), that were the reasons for their visit. A total of 11,100 patients, of whom 5474 (49.3%) were female, were examined over a 6-year period. The median patient age was 70 years (range, 1–101 years) for the entire period; 1.4% of the examinees were under 20 years old, 23.5% were between 20 and 59 years old, and 75.1% were 60 years old or older. During the examination period, 1491 persons were examined in the 2 years and 3 months before the evacuation order lifted on June 2016, of whom 504 (33.8%) were female. After lifting the evacuation order, a total of 9609 persons were examined over a period of 3 years and 9 months, of whom 4970 (51.7%) were female. The monthly average of the number of persons seen before and after the lifting of the evacuation order increased by 158.3 persons, from 55.2 to 213.5. A comparison of the percentage of female patients receiving medical examinations showed that their percentage increased by 17.9%, from 33.8% to 51.7%. As per age, those aged under 20 years old increased by 0.6%, from 0.8% to 1.4%; those aged 20 to 59 years old decreased by 19.9%, from 40.7% to 20.8%; and those aged 60 years old and older increased by 19.2%, from 58.5% to 77.7%.

**Table 3:** Details of the study population.

	Before lifting the evacuation order			After lifting the evacuation order				Total
	2014/4–2015/3	2015/4–2016/3	2016/4–2016/6	2016/7–2017/3	2017/4–2018/3	2018/4–2019/3	2019/4–2020/3	
Living in Odaka ward*	0	0	0	310	1487	2639	3491	
Total patients	459	811	221	1181	2374	2763	3291	11,100
Living area of patients								
Odaka ward	257 (56.0)	387 (47.7)	154 (69.7)	674 (57.1)	1706 (71.9)	2148 (77.7)	2796 (85.0)	8122 (73.2)
MC except Odaka ward	64 (13.9)	134 (16.5)	40 (18.1)	196 (16.6)	349 (14.7)	310 (11.2)	255 (7.7)	1348 (12.1)
Fukushima Pref. except MC	40 (8.7)	72 (8.9)	11 (5.0)	126 (10.7)	132 (5.6)	145 (5.2)	129 (3.9)	655 (5.9)
Other Pref.	98 (21.4)	218 (26.9)	16 (7.2)	184 (15.6)	178 (7.5)	160 (5.8)	111 (3.4)	965 (8.7)
Unknown	0 (0.0)	0 (.0)	0 (.0)	1 (.1)	9 (.4)	0 (.0)	0 (.0)	10 (.1)
Male	297 (64.7)	561 (69.2)	129 (58.4)	722 (61.1)	1174 (49.5)	1245 (45.1)	1498 (45.5)	5626 (50.7)
Female	162 (35.3)	250 (30.8)	92 (41.6)	459 (38.9)	1200 (50.5)	1518 (54.9)	1793 (54.5)	5474 (49.3)
Age (yr)								
≤19	6 (1.3)	4 (.5)	2 (.9)	13 (1.1)	54 (2.3)	44 (1.6)	28 (.9)	151 (1.4)
20–29	16 (3.5)	24 (3.0)	6 (2.7)	44 (3.7)	49 (2.1)	38 (1.4)	26 (.8)	203 (1.8)
30–39	35 (7.6)	59 (7.3)	9 (4.1)	64 (5.4)	84 (3.5)	50 (1.8)	60 (1.8)	361 (3.3)
40–49	60 (13.1)	103 (12.7)	18 (8.1)	127 (10.8)	157 (6.6)	159 (5.8)	124 (3.8)	748 (6.7)
50–59	67 (14.6)	172 (21.2)	38 (17.2)	198 (16.8)	291 (12.3)	246 (8.9)	284 (8.6)	1296 (11.7)
60–69	161 (35.1)	266 (32.8)	77 (34.8)	310 (26.2)	524 (22.1)	560 (20.3)	623 (18.9)	2521 (22.7)
70–79	95 (20.7)	147 (18.1)	45 (20.4)	229 (19.4)	575 (24.2)	662 (24.0)	906 (27.5)	2659 (24.0)
≥80	19 (4.1)	36 (4.4)	26 (11.8)	196 (16.6)	640 (27.0)	1004 (36.3)	1240 (37.7)	3161 (28.5)
Mean	59.2	59.3	62.8	62.4	67.1	71	72.8	68.4
Median	63	62	66	65	70	74	75	70
Range	9-88	1-97	3-91	5-94	1-101	3-99	3-100	1-101
First visit†	209 (45.5)	347 (42.8)	72 (32.6)	490 (41.5)	723 (30.5)	488 (17.7)	529 (16.1)	2858 (25.7)
Following treatment	250 (54.5)	464 (57.2)	149 (67.4)	691 (58.5)	1651 (69.5)	2275 (82.3)	2762 (83.9)	8242 (74.3)
Emergency case	8 (1.7)	10 (1.2)	2 (.9)	5 (.4)	1 (.0)	4 (.1)	3 (.1)	33 (.3)
Patients with								
Musculoskeletal diseases	110 (24.0)	190 (23.4)	55 (24.9)	153 (13.0)	288 (12.1)	379 (13.7)	433 (13.2)	1608 (14.5)
	355 (23.8)			1253 (13.0)				
Psychiatric diseases	44 (9.6)	72 (8.9)	22 (10.0)	85 (7.2)	212 (8.9)	524 (19.0)	662 (20.1)	1621 (14.6)
	138 (9.3)			1483 (15.4)				
Injuries or external causes	58 (12.6)	144 (17.8)	11 (5.0)	120 (10.2)	130 (5.5)	63 (2.3)	62 (1.9)	588 (5.3)
	213 (14.3)			375 (3.9)				

MC = Minamisoma city.

* The numbers in the table for each term represent the numbers as of the last day of the respective previous term. For the term 2016/7–2017/3, the numbers are as of 2016/7/31, which is immediately after the evacuation order was lifted.

†“ First visit” means the first consultation in this research period. If they had visited Odaka Hospital before the earthquake, that visit was not taken into consideration.

**Table 4:** The top disease names and codes of ICD-10 that were the reasons for the patients’ visit.

	Before lifting the evacuation order			After lifting the evacuation order		
	ICD-10 Code	Disease name	N (%)	ICD-10 Code	Disease name	N (%)
1	I10	Essential (primary) hypertension	402 (27.0)	I10	Essential (primary) hypertension	5483 (57.1)
2	E78	Disorders of lipoprotein metabolism and other lipidemias	193 (12.9)	E78	Disorders of lipoprotein metabolism and other lipidemias	2804 (29.2)
3	J00	Acute nasopharyngitis [common cold]	138 (9.3)	F51	Sleep disorders	1073 (11.2)
4	M53	Other and unspecified dorsopathies, not elsewhere classified	135 (9.1)	E11	Type 2 diabetes mellitus	1008 (10.5)
5	M54	Dorsalgia	125 (8.4)	K59	Other functional intestinal disorders	657 (6.8)
6	T63	Toxic effect of contact with venomous animals and plants	106 (7.1)	K21	Gastro-esophageal reflux disease	584 (6.1)
7	M17	Osteoarthritis of knee	101 (6.8)	J02	Acute pharyngitis	555 (5.8)
8	F51	Sleep disorders	92 (6.2)	K29	Gastritis and duodenitis	431 (4.5)
9	E11	Type 2 diabetes mellitus	87 (5.8)	E79	Disorders of purine and pyrimidine metabolism	429 (4.5)
10	T67	Heatstroke and sunstroke	56 (3.8)	M54	Dorsalgia	392 (4.1)
11	K29	Gastritis and duodenitis	55 (3.7)	I69	Sequelae of cerebrovascular disease	391 (4.1)
12	J9-11	Influenza	46 (3.1)	M53	Other and unspecified dorsopathies, not elsewhere classified	386 (4.0)
13	I20	Angina pectoris	44 (3.0)	J20	Acute bronchitis	372 (3.9)
14	K21	Gastro-esophageal reflux disease	44 (3.0)	J30	Vasomotor and allergic rhinitis	364 (3.8)
15	J30	Vasomotor and allergic rhinitis	42 (2.8)	J9–11	Influenza	331 (3.4)
16	E02-03	Hypothyroidism	31 (2.1)	K25	Gastric ulcer	298 (3.1)
17	M48	Other spondylopathies	29 (1.9)	F00-03	Dementia	294 (3.1)
18	S80	Superficial injury of knee and lower leg	27 (1.8)	J45	Asthma	195 (2.0)
19	F32–34	Major depressive disorder, single episode	23 (1.5)	M17	Osteoarthritis of knee	193 (2.0)
20	J02	Acute pharyngitis	23 (1.5)	I48	Atrial fibrillation and flutter	181 (1.9)

ICD-10 = International Classification of Diseases, Tenth Edition.

During the entire period, 25.7% of the patients underwent one visit and 74.3% received a following treatment. By period, 42.1% of the patients received an initial visit, and 57.9% received a following treatment, before the evacuation order was lifted. Subsequently, 23.2% of the patients received an initial visit, and 76.8% a following treatment, after the evacuation order was lifted. Before and after the evacuation order was lifted, initial consultations decreased by 18.9%, from 42.1% to 23.2%, whereas return visits increased by 18.9%, from 57.9% to 76.8%.

Before the evacuation order lifted, 53.5% of the participants lived in Odaka Ward, 16.0% in Minamisoma City other than Odaka Ward (Haramachi-ku and Kashima-ku), 8.2% in Fukushima Prefecture other than Minamisoma City, and 22.3% in other prefectures. After the evacuation order was lifted, the percentages of participants from Odaka Ward, Minamisoma City, Fukushima Prefecture, and other prefectures increased to 76.2%, 11.6%, 5.5%, and 6.6%, respectively.

In a comparison with the health status of the group with a disease at the time of consultation, before and after the lifting of the evacuation order, musculoskeletal diseases decreased by 10.7%, from 23.8% to 13.0% (χ square test, χ^2^(1) = 93.6, *P* < .01), psychiatric diseases increased by 6.2%, from 9.3% to 15.4% (χ^2^(1) = 39.5, *P* < .01), and diseases due to trauma or external causes decreased by 10.4%, from 14.3% to 3.9% (χ^2^(1) = 277.4, *P* < .01). Hypertension (53.0%), dyslipidemia (27.0%), insomnia (10.5%), and Type 2 diabetes mellitus (9.9%) were the most common diseases during the study period. Before and after the lifting of the evacuation order, hypertension was the most common (27.0%, 57.1%), followed by dyslipidemia (12.9%, 29.2%). Acute nasopharyngitis [common cold] (9.3%), other and unspecified dorsopathies, not elsewhere classified (9.1%), ranked third and fourth before lifting of the evacuation order, respectively. After lifting of the evacuation order, the third and fourth most common were sleep disorders (11.2%) and Type 2 diabetes mellitus (10.5%), respectively. When classified by the time of visit, before lifting of the evacuation order, illnesses caused by trauma and external causes, including the toxic effects of contact with venomous animals and plants (1.0%) and heatstroke and sunstroke (0.5%), were noted. After the evacuation order was lifted, noncommunicable diseases (NCDs), such as hypertension (57.1%), dyslipidemia (29.2%), insomnia (11.2%), and Type 2 diabetes mellitus (10.5%), ranked high.

Of all the consultations, 33 (0.3%) of the participants were considered urgent cases, and were transported by ambulance or referred to other hospitals. At the time of consultation, 20 of 1491 (1.3%) were considered urgent cases before the evacuation order was lifted, while 13 of 9609 (0.1%) were considered urgent cases after the evacuation order was lifted. The proportion of urgent cases decreased by 1.2%, from 1.3% to 0.1%, before and after the evacuation order was lifted. The main reasons for transportation were anaphylactic shock due to bee stings and heatstroke.

## 4. Discussion

This study examined the population of patients and reasons for their clinic visits in areas where evacuation orders were lifted after a radiation disaster. Our results highlight the importance of primary care in these areas to ensure the effective use of limited medical resources. Primary care, according to Starfield, is characterized by first contact care, longitudinally, comprehensive care, and coordinated care.^[25]^ These characteristics are consistent with the medical needs of evacuation-designated areas in the aftermath of a radiation disaster.

First, our results highlight the diverse reasons for consultation and the need for long-term therapies, both of which support the importance of primary care. Specifically, Table 4 shows that in addition to NCDs, such as hypertension, dyslipidemia, and type 2 diabetes, psychiatric disorders (insomnia, depression, and other ICD-10 F codes) and “musculoskeletal disorders other than trauma” were common during the entire period. In addition, diseases caused by trauma and external causes, including heatstroke and toxic effects of contact with venomous animals and plants, was noted before the evacuation order was lifted. As previously reported,^[26]^ bee sting illnesses associated with restoration work are frequently associated with the toxic effects of contact with venomous animals and plants.

Chronic diseases requiring long-term therapies were also more common during the disaster recovery period.^[27]^ Before lifting of the evacuation order, the main cause of hospital visits was NCDs, and emergency patients were few. This trend became even more pronounced after the evacuation order was lifted; specifically, psychiatric and non-traumatic musculoskeletal disorders increased, NCDs and insomnia ranked higher, and the proportion of emergency cases decreased. This trend is consistent with a report on the medical demands in Kawauchi Village,^[28–30]^ the area to which people returned after the GEJE. Considering that the return to the evacuation zone remains in progress, especially among older individuals,^[31]^ and that the population in the evacuation zone will continue to age, the number of people who will require long-term support is expected to continue to increase in the future. Thus, many diverse diseases and long-term therapies are required.^[32]^

Second, it is necessary to respond to changing medical needs. As of 2020, the aging rate of residents returning to areas where evacuation orders have been lifted was 49%. Furthermore, the average age of the patients at Odaka Hospital was also relatively high, at 68.4 years old. Notably, this generation must live with and come to terms with multiple diseases.^[33]^ To support the older people, who have returned to areas where evacuation orders have been lifted as their final home until their death, social support, such as nursing care services and home medical care,^[34]^ is needed to enable the individuals to live well, even after their physical abilities (mobility) have deteriorated. At the Odaka Hospital, the average age of patients is expected to increase further in the future, and the clinic needs to expand its medical care delivery system to meet the needs of older patients, including home medical care.^[35–37]^

Third, increased medical needs and consultations with populations at high health risk have been observed. Such needs require the effective use of limited medical resources in areas where evacuation orders have been lifted as well as the long-term control of medical needs.

The results show that the number of persons seen and medical needs in the areas where evacuation orders were lifted both gradually increased. Specifically, Table 3 shows that the average monthly number of persons seen increased by 158.3 persons, from 55.2 to 213.5 persons, before and after the lifting of evacuation orders. The total number of annual visits also increased consistently after the evacuation order was lifted, from 1181 (131.2 visits/mo) from July 2016 to March 2017 to 3291 (274.3 visits/mo) from April 2019 to March 2020. As stated by Sawano et al,^[38]^ decontamination workers are widely recruited from outside the prefecture; thus, it can be assumed that the decontamination workers mainly accounted for those living outside the prefecture. The percentage of out-of-prefecture residents among the examinees decreased, whereas that of Odaka residents increased, suggesting a shift in the examinee population from temporary workers to local residents.

After the GEJE, the patients who visited Odaka Hospital included decommissioning and decontamination workers. Reportedly, decontamination workers have a high prevalence of NCDs, as well as high rates of alcohol consumption, smoking, and obesity,^[38]^ making them a group with high health risks. As presented in the previous section, changes in the group of examinees can be pointed out along the time series; however, all of them are at high health risk; thus, the limited medical resources need to be used effectively. It is necessary to consider the backgrounds of both the workers and the older people and to implement a comprehensive approach to improve their health.

## 5. Limitations

This study has some limitations. First, there is a lack of comparison between the age distribution of the residential population in Odaka and that of the medical examiners. The percentage of people receiving medical examinations in each age group of the resident population is necessary for estimating medical demand and public health practices; however, this information is lacking in this study. Notably, in areas where evacuation orders were lifted, it was difficult to determine the actual resident population in the first place.

Second, no data were available for the period before the GEJE. Although the reasons for medical treatment in this area before the GEJE may have been similar to those after the evacuation order was lifted, it was not possible to compare the situations before and after the GEJE. Enter third, to use the results of this study to mitigate devastation from future disasters, it is necessary to consider the factors unique to the Odaka Ward, Minamisoma City, compared to other areas. For example, the backgrounds of the decontamination workers differed. While most of the decontamination workers involved in the Chornobyl disaster were military personnel,^[39]^ most of the workers involved in this recent disaster were migrant workers, with low socioeconomic status.^[40]^ Furthermore, the fact that most returnees were older individuals, pose a different situation compared to that of other disasters. Additionally, during the survey period, those living in the area at the time of the GEJE were exempt from medical expenses, which may have stimulated their demand for medical care. These points should be considered when applying the results of this study to other disasters.

In conclusion, we found that in evacuation-designated areas, the need for primary care increased due to the existence of diverse diseases, ranging from trauma to NCDs and psychiatric disorders, as well as medical demands that required long-term therapies. Moreover, the demand for medical care changed over time, as the main group of patients shifted from those moving out of Minamisoma City to returning residents, before and after the lifting of the evacuation order. Thus, effective utilization of medical resources is necessary. Based on the characteristics of the medical demand in areas where evacuation orders have been lifted, it is desirable to develop a health maintenance system.

## Acknowledgements

We would like to thank the study director along with the doctors and nurses of Odaka Hospital who were involved in the medical treatments related to our study. The authors are also grateful to Mr Masatsugu Tanaki of Minamisoma Municipal General Hospital for his technical support. Additionally, the authors thank Editage (www.editage.com) for English language editing.

## Author contributions

**Conceptualization:** Saori Nonaka, Masaaki Odaka, Masaharu Tsubokura.

**Data curation:** Saori Nonaka, Yuki Senoo.

**Formal analysis:** Saori Nonaka.

**Writing – original draft:** Saori Nonaka, Masaharu Tsubokura.

**Writing – review & editing:** Masaaki Odaka, Toyoaki Sawano, Akihiko Ozaki, Michio Murakami, Makoto Yoshida, Yuna Uchi, Katsuko Onoda, Tomoyoshi Oikawa.

**Investigation:** Akemi Takada.

**Funding acquisition:** Masaharu Tsubokura.

**Project administration:** Masaharu Tsubokura.
